# Establishing a unified global framework for studying dementia knowledge: insights from a narrative review

**DOI:** 10.1186/s13690-024-01476-1

**Published:** 2024-12-23

**Authors:** Sophia Lazarova, Dessislava Petrova-Antonova

**Affiliations:** 1 GATE Institute, Sofia University “St. Kliment Ohridski”, Sofia, 1504 Bulgaria; 2 Faculty of Mathematics and Informatics, Sofia University “St. Kliment Ohridski”, Sofia, 1504 Bulgaria

**Keywords:** dementia knowledge, survey framework, public health, dementia prevention, dementia education, dementia literacy

## Abstract

**Background:**

With the global population aging rapidly, dementia has become a pressing public health challenge, affecting the cognitive functions and daily activities of older adults worldwide. Enhancing literacy about dementia is a proactive prevention strategy, yet the effectiveness of educational programs can vary based on the target population’s background. Thus, understanding dementia knowledge levels across different communities and countries is essential for successful educational interventions. Despite the large аmount of studies, there is no common framework for studying dementia knowledge, leading to significant variability in methods and poor data comparability.

**Methods:**

A narrative review is conducted to examine the methodological variability in studies of dementia knowledge and to propose a unified framework for future investigations. We hypothesize that significant differences will be evident in the methodologies employed, particularly regarding knowledge domains, research designs, influencing factors, and assessments of attitudes toward dementia.

**Results:**

A total of 59 research publications published after 2000 were selected, revealing significant variability in approaches to studying dementia knowledge and confirming our hypothesis. We identified eight dementia knowledge domains and various sociodemographic and experiential correlates, along with commonly used complementary assessments. These findings were organized into a unified global framework comprising two core components—dementia knowledge domains and correlates—supplemented by a component addressing affective dispositions towards dementia and an action list to guide future research. The framework aims to provide a foundational basis for enhancing inter-study comparisons and deepening our understanding of dementia knowledge and attitudes across diverse communities.

**Conclusion:**

Aligning methodologies for surveying dementia knowledge through a common framework can empower stakeholders to implement effective educational programs, fostering an informed and supportive environment for individuals affected by dementia.

**Supplementary Information:**

The online version contains supplementary material available at 10.1186/s13690-024-01476-1.

**Table:** 

**Text box 1. Contributions to the literature**
• There is significant methodological variability in conducting studies on dementia knowledge, leading to difficulties in data comparability.
• Variabilities exist across several aspects: (1) surveyed knowledge domains, (2) instruments used for assessment, (3) factors influencing dementia knowledge, and (4) inclusion of complementary assessments of attitudes towards dementia.
• This work proposes a practice-based, unified framework for conducting studies on dementia knowledge.

## Introduction

Dementia is a leading cause of dependency among older adults, progressively impairing cognitive functions and behavior. With nearly 9.9 million new dementia cases diagnosed annually—equating to one every three seconds [1], the urgency to address this growing crisis is clear.

In addition to its profound physical and psychological impacts, dementia imposes significant financial burdens on governments and families. In 2015, the costs associated with dementia were estimated at US$ 818 billion, and by 2030, this figure is projected to escalate to US$ 2 trillion, potentially overwhelming health and social services [2]. Given these projections, alongside the immense financial, social, and emotional toll, it is evident that dementia represents a persistent health crisis affecting millions worldwide. Until effective treatments are discovered, promoting disease prevention and limiting its progression through informed educational initiatives remains a critical priority.

### Prevention and dementia literacy

Primary prevention focuses on health promotion and mitigation of risk factors as means to avoid the onset of a disease or adverse events before they occur [3]. As a part of primary prevention, health promotion involves activities such as health education, risk factor awareness, and promotion of healthy and risk-lowering behaviors [3]. Therefore, health literacy, defined as the ability to access, process, and understand health information, is crucial for disease prevention. A systematic review from 2019 reported a positive association between health literacy scores and the post-mortem amount of plaques and tangles in Alzheimer’s disease patients suggesting that low levels of health literacy might lead to higher future dementia risk [4]. Furthermore, educational interventions were shown to significantly improve knowledge and preventive behaviors in various health contexts, from infectious diseases to cardiovascular health [5–9]. For example, a study from 2021 found а decrease in body-mass index, improved control of diabetes and lipid profiles, improved knowledge as well as a gradual transition to a plant-based diet after a 3-month education, marking а significant difference in the preventive behaviors of the group [10].

Although dementia is currently incurable, about 40% of worldwide dementias are attributed to modifiable risk factors and could theoretically be prevented or delayed through lifestyle changes [11]. On one hand, increasing dementia literacy is a key aspect of enabling individuals to engage in risk-lowering behaviors. On the other, effective informational outreach is heavily dependent on tailoring educational efforts to cultural contexts and the current state of regional knowledge. Thus, studies of dementia knowledge are vital for elevating health literacy as they serve a double purpose – (1) they inform the development of educational programs by presenting the state of current knowledge and (2) they are used to examine the effectiveness of educational programs or other public outreach campaigns.

### The problem of methodological inconsistency

While dementia knowledge has been studied for several decades now, there are no established frameworks navigating the design of such studies. As a result, even though numerous studies have been conducted, their results remain largely incomparable. A systematic review, conducted in 2015, reported significant inconsistencies in dementia knowledge research regarding operationalizing dementia knowledge, sampling methods, response rates, and data collection tools [12]. Other studies also have noted the substantial variability in the methods used to study dementia knowledge [13–15]. These methodological challenges create difficulties in comparing misaligned data, underscoring the need for improved data interoperability. Aligning survey protocols will facilitate a comprehensive understanding of dementia awareness determinants and support the development of evidence-based educational programs.

### Towards a unified account of studying dementia knowledge

The present study proposes a framework aimed at aligning existing methods for studying dementia knowledge. The framework is based on qualitative analyses of existing dementia knowledge studies, providing a structured approach for future research. By exploring the methodological approaches of previous works, we outline a unified account of creating structured and informative dementia knowledge studies. Based on practice, the framework aims to align research methodologies, facilitate comparisons across studies, and advance the current understanding of public dementia knowledge.

We hypothesize that significant variability will be observed in terms of knowledge domains, survey methodologies, factors influencing literacy, and attitude assessments. Assessing attitudes is crucial, as personal dispositions can impact both knowledge acquisition and the willingness to seek help. A strong connection between attitudes and knowledge processing and acquisition has been documented in phenomena such as confirmation bias and cognitive dissonance theory [16, 17]. Furthermore, previous studies on knowledge, perceptions, and disease have confirmed the importance of personal attitudes in knowledge acquisition and help-seeking [18–21]. Thus, our framework aims to address all critical aspects of knowledge studies, establishing a common theoretical structure for dementia research.

The paper is organized as follows: Sect. 2 outlines the methods used for literature review and framework development, Sect. 3 presents insights and a unified framework based on existing literature, Sect. 4 discusses results and implications, Sect. 5 addresses limitations and future research opportunities, and Sect. 6 concludes the study.

## Materials and methods

This work proposes a unified framework for studying dementia knowledge, informed by the variability in existing methodological approaches to communal literacy. We analyze current methods for studying dementia knowledge to ensure the framework’s relevance and applicability. By applying a practice-informed approach, we aim to enhance the framework’s adoption and real-world application.

### Literature review

An empirical integrative review approach was used to integrate a variety of methodologies and obtain a holistic understanding of the research strategies applied to dementia knowledge studies. This type of narrative approach was considered appropriate as it allows in-depth analyses of methodological issues within a particular topic [22, 23]. To increase the rigor of the present work, we followed the five-stage methodology for integrative reviews proposed by Whittemore and Knafl [23] and elements of the PRISMA guidelines [24].

### Literature search

We conducted a literature search in three databases: PubMed, Web of Science, and Scopus, using the following keywords ‘dementia knowledge’, ‘dementia understanding’, ‘dementia awareness’, ‘dementia literacy’ as well as variations using ‘Alzheimer’s disease’ instead of ‘dementia’. Results were filtered by publication year (January 2000 – July 2024), article type (research), and language (English), excluding grey literature, abstracts, and secondary sources.

### Literature screening

The titles and abstracts of all articles retrieved from electronic databases were screened for appropriateness. Only research articles that studied at least one aspect of dementia knowledge were considered for further analysis. No restrictions were imposed on the type and size of the studied samples. Similarly, there were no restrictions on the country of origin. Articles evaluating dementia knowledge assessment tools or articles assessing educational or awareness campaigns were not considered. Similarly, research articles focusing on highly specialized aspects of dementia knowledge related to professional background were not considered.

### Literature evaluation and analysis

The full texts of all relevant items were carefully reviewed and analyzed. The methodology of each article was evaluated in terms of the following aspects: (1) surveyed dimensions of dementia knowledge, (2) methods for studying dementia knowledge, (3) considered correlates of dementia knowledge and (4) complementary assessments. Obtained results are presented with percentages and observed methodological variabilities are structured and discussed thoroughly. Note that the present work does not discuss the findings and results produced by the reviewed literature. Instead, it focuses on the methods employed to study dementia knowledge in order to evaluate their strengths and weaknesses and compile a framework for design of future studies.

### Framework development

We developed a framework for studying dementia knowledge based on insights from the identified methodological variability in existing literature. These synthesized observations informed the formulation of the framework, its components as well as a checklist of activities, designed to navigate the planning stages of dementia knowledge studies.

## Results

### Search results

**Fig. 1:**
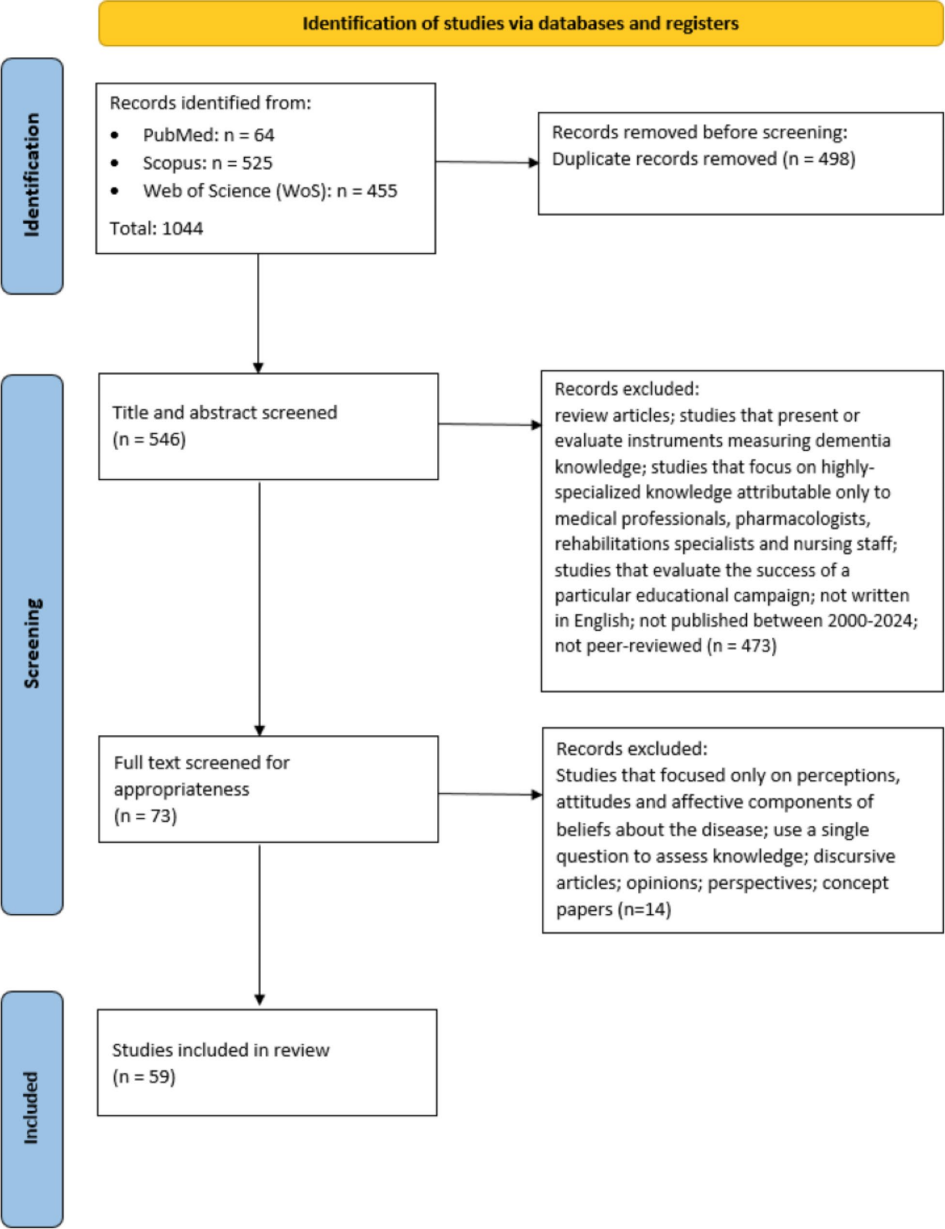
PRISMA flow diagram and selection of relevant articles

A total of 1044 articles were retrieved via database searching. After removing duplicates and screening for appropriateness, a total of 59 publications were determined relevant (Table S1 Additional File 1). A detailed search and selection strategy is presented in Fig. 1.

### Characteristics of relevant literature

**Fig. 2:**
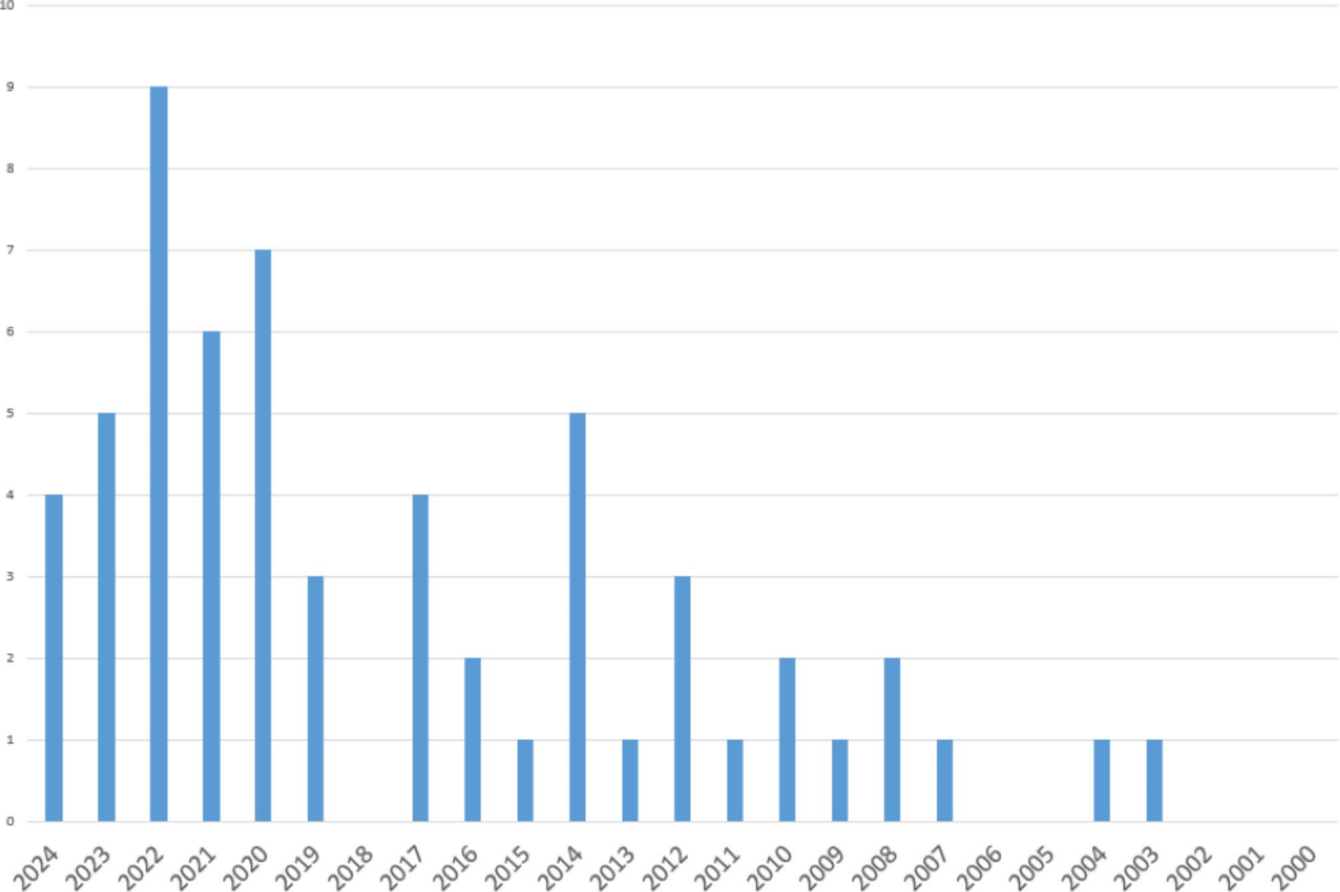
Distribution of the selected papers according to year of publication

More than half of the selected articles were published in the last five years, thus representing the latest research in the field of dementia knowledge while also capturing research published in earlier time periods (Fig. 2).

**Fig. 3:**
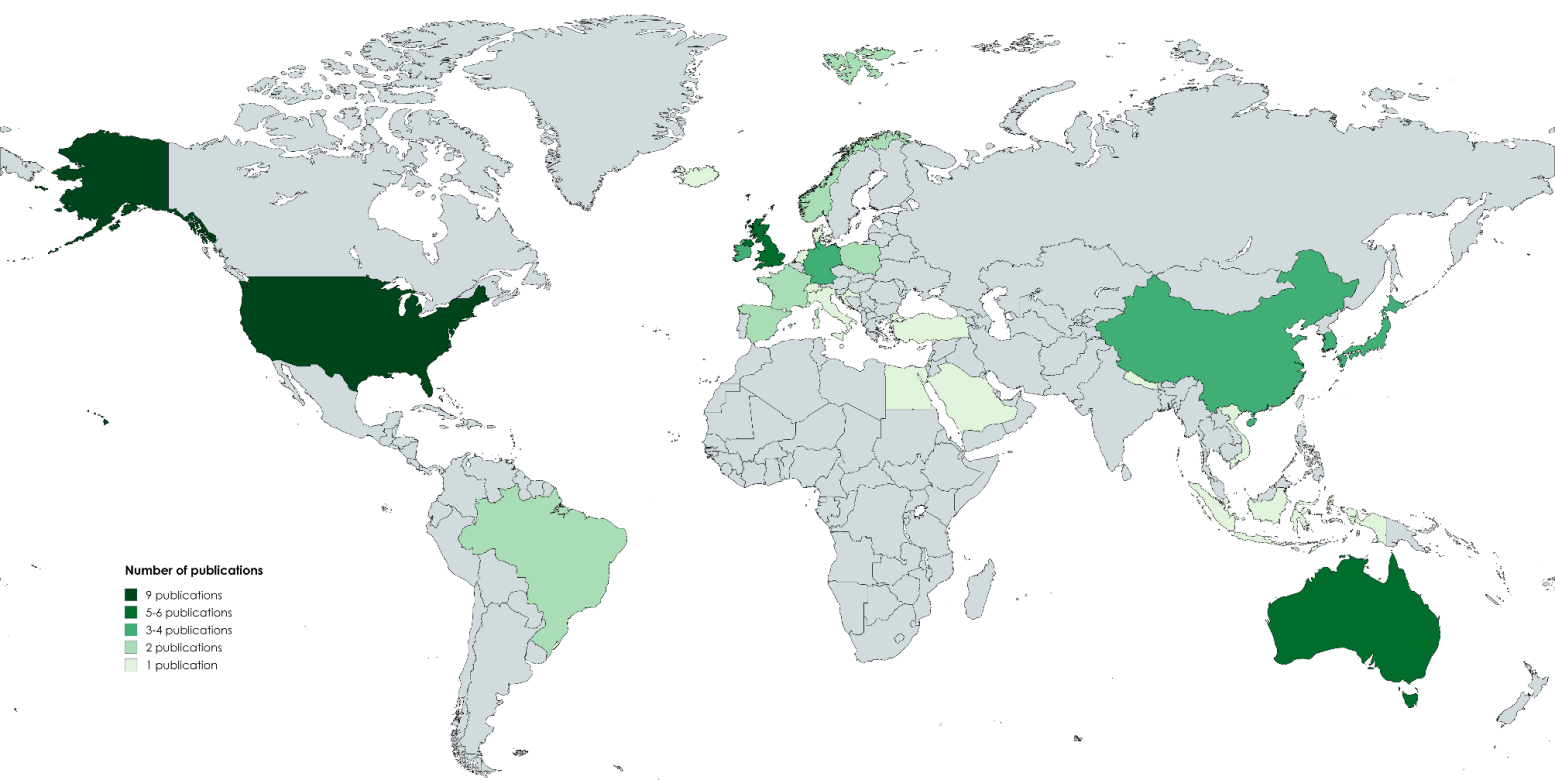
A Color-coded map depicting the number of publications per country

The final literature sample spans over twenty-eight countries across five continents, with the European region showing the highest representation (Fig. 3). The USA leads with nine publications, followed by Australia and the UK. Notably, dementia knowledge is significantly less explored in low- and middle-income regions than in high-income countries, a trend that has persisted over time, as highlighted in a 2018 systematic review [25].

### Observed methodological variability in studies of dementia knowledge

The literature revealed significant variability in approaches to studying dementia knowledge, confirming our hypothesis. Differences emerged regarding the definition of ‘dementia knowledge,’ survey methodologies, and factors hypothesized to influence knowledge. The rest of this section offers an analysis of the observed characteristics of existing research articles on communal dementia knowledge.

### Dementia knowledge as a multi-domain construct

Dementia knowledge refers to the beliefs and information individuals possess about the disease. Thus, it can be described as a multi-domain construct in which the different domains have varying levels of importance depending on the target group. Our analysis identified eight key domains of dementia knowledge: (1) General Knowledge, (2) Etiology, (3) Epidemiology, (4) Disease Course and Life Impact, (5) Assessment and Diagnosis, (6) Symptoms, (7) Care and Management, and (8) Risk Factors and Protective Behaviors. “General knowledge” is defined as non-specific knowledge about dementia that conveys general facts about the condition usually considered as “common knowledge”. Some questions that fall in the category of “general knowledge” would be “Which part of the body is primarily affected by dementia?”, “Is dementia curable?”, “Is dementia a normal part of aging?”.

**Fig. 4:**
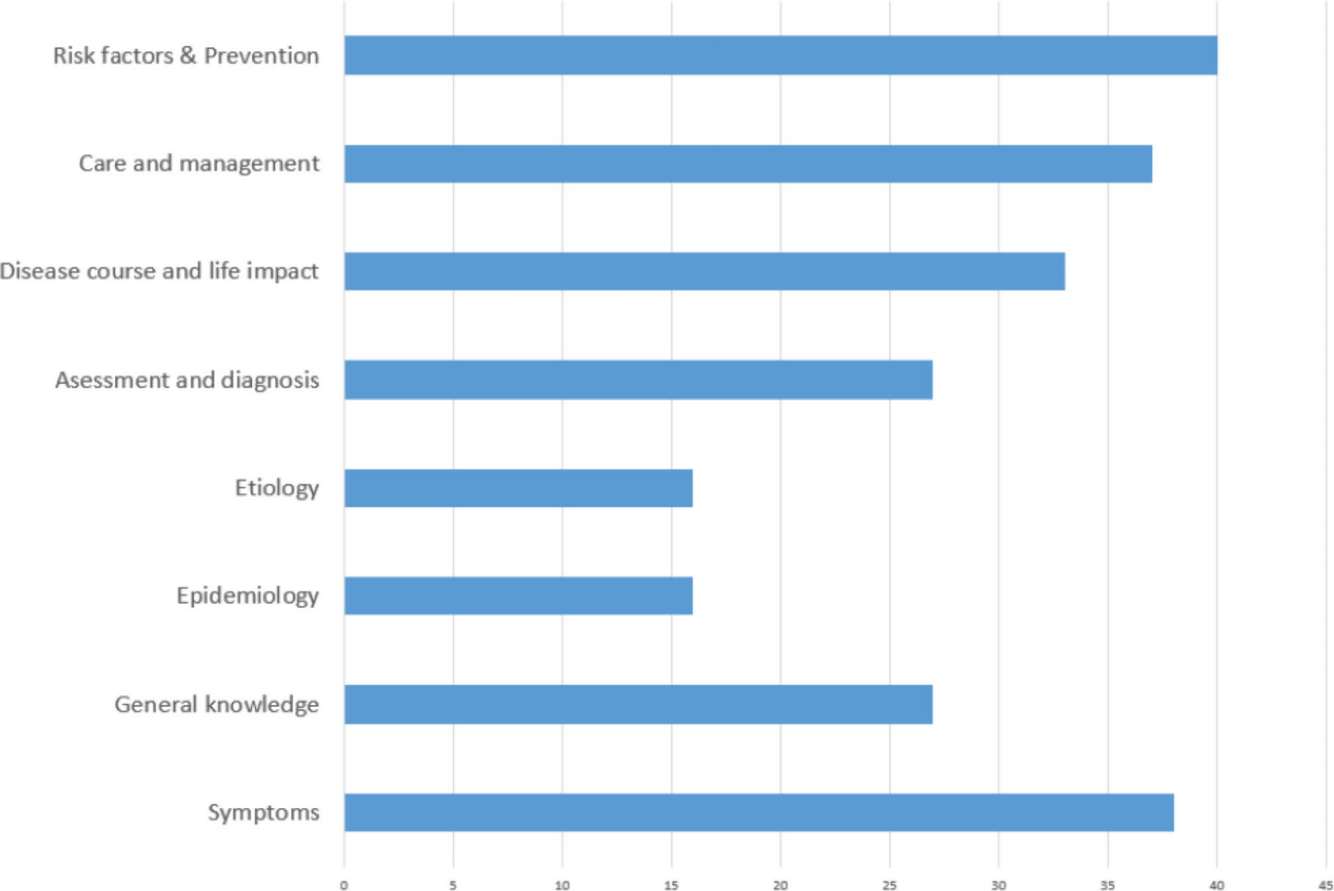
Number of publications addressing each of the dementia knowledge domains

The most frequently addressed domains were ‘Risk Factors and Prevention,’ followed closely by ‘Symptoms’ and ‘Care and Management.’ Conversely, ‘Etiology’ and ‘Epidemiology’ received less attention, particularly in studies involving non-specialized populations (Fig. 4). These results reflect the social importance of risk factors and symptoms literacy for the general population. Conversely, domains representing specialized knowledge (etiology, epidemiology, diagnosis) are more relevant to medical professionals.

### Multi-domain surveys of dementia knowledge

#### Approaches to multi-domain dementia knowledge surveys

This section outlines several approaches to surveying dementia knowledge: (1) reusing validated instruments, (2) partially reusing validated instruments, and (3) creating custom instruments. The appropriateness of each approach depends on several factors including data acquisition methods, target sample characteristics, study length, and required depth of knowledge.

#### On the use of validated multi-domain instruments for surveying dementia knowledge

Validated multi-domain instruments are questionnaires designed to assess various aspects of dementia knowledge, including at least one item per domain. These instruments are convenient due to their proven efficacy and compatibility with data from previous studies. However, their comprehensive nature often results in lengthy questionnaires, which can be burdensome for participants, especially during telephone surveys or as part of larger studies. While aiming to quantify overall dementia knowledge, these instruments may provide insufficient depth in each domain, leading to potential ceiling effects in more knowledgeable groups. The variability among instruments can be illustrated by comparing the most commonly used multi-domain scales within our literature sample, such as the Alzheimer’s Disease Knowledge Scale (ADKS) [13], the Dementia Knowledge Assessment Scale (DKAS) [15, 26], the Dementia Knowledge Questionnaire (DKQ) and finally, the Dementia Knowledge Assessment Tool (DKAT2) [27] (Table 1). For each instrument, differences can be observed in the number of questions, response formats, domain coverage, and content relevance.

**Table 1:** Brief description of the most used dementia knowledge scales among the selected publications (diverse population). Knowledge domains are numerically represented under the following mapping: (1) general knowledge; (2) etiology; (3) epidemiology; (4) disease course and life impact; (5) assessment and diagnosis; (6) symptoms; (7) care and management and finally; (8) risk factors and protective behaviors

Scale	Year	Items	Response	Domains	Features	Limitations
Alzheimer’s Disease Knowledge Scale (ADKS)	2009	30	Yes/No	4;5;6;7;8	- Suitable for varied populations. - Completed for 10–15 min. - Commonly used. Possible result comparisons. - Broad domain coverage. - Can detect changes pre- and post- education.	- Prone to ceiling effects especially in more expert groups. - Inconsistent reliability.
Dementia Knowledge Assessment Scale (DKAS)	2015	25	5-item Likert scale (from False to True with auxiliary option I don’t know)	1;2;3;4;7;8	- Broad domain coverage. - Lower ceiling effect even in more knowledgeable groups. - Developed and tested on a large and diverse sample. - Better discrimation pre- and posteducation compared to ADKS [28]	
Dementia Knowledge Questionnaire (DKQ)	1997	7	Five questions with one correct answer, two questions with lists where each item is answered Yes/No/I don’t know	1;2;3;6;8	- Short and simple. - Several domains of knowledge.	- Limited domain depth coverage. - No update since 1997. - No evaluation of psychometric properties.
Dementia Knowledge Assessment Tool (DKAT2)	2014	21	Yes/No/I don’t know	1;2;3;4;6;7	- Broad domain coverage. - Developed to measure knowledge in the adult population.	- Possible ceiling effects - Inferior psychometric properties compared to ADKS and DKAS

We observed a preference towards ADKS and DKAS compared to other scales within the sampled literature. This observation is in line with previous studies as a systematic literature review evaluated the psychometric quality of four dementia knowledge scales (ADKS, DKAS, DKAT2 and Dementia Knowledge 20 (DK-20) [28]) and confirmed the superior psychometric properties of ADKS and DKAS compared to the other two scales [29]. The similarity in performance between ADKS and DKAS is not a coincidence as there is a level of correlation between the two scales, suggesting that both measure a relevant knowledge construct [30]. However, ADKS seems inferior to DKAS in terms of ceiling effect due to the dichotomous format of the responses (True/False), especially in more knowledgeable samples. Furthermore, DKAS has better construct validity and is shown to perform better in large cohorts [30].

In summary, reusing validated instruments is strongly encouraged due to the demonstrated efficacy of the instruments and the ensured comparability with other studies using the same instrument. However, several caveats need to be considered before settling on an existing dementia knowledge instrument. First, while many dementia knowledge scales cover a relatively broad scope of knowledge domains, the depth of the examined knowledge may substantially vary between instruments. Furthermore, dementia knowledge scales vary in terms of specialization, psychometric qualities, length, and complexity. Next, the availability of particular scales may pose additional difficulties in reusing existing instruments. For instance, obtaining permission for use is often a lengthy process and in some cases acquiring permission may prove to be impossible, thus creating an additional set of obstacles for researchers. Another caveat worth consideration is the cultural variation occurring across communities and nations. Cultural variations may render instruments inapplicable to certain groups, thus the choice of a validated instrument should be guided by the characteristics of the studied population. Validating the psychometric properties of existing instruments in various populations is a crucial step in limiting the possible implications of this issue. Similarly, when translation or adaptations are imperative one should ensure that the properties of the instrument remain intact and it still measures the intended constructs.

Finally, dementia knowledge scales reflect the current body of knowledge relevant to the time of their development. Therefore, if not periodically revised, existing knowledge scales may become outdated or fail to incorporate the latest advancements in science and medicine. These variations highlight the need for careful consideration when selecting an instrument for dementia knowledge assessment.

#### Partially reusing existing multi-domain instruments

Validated multi-domain instruments can be adapted to meet the specific needs of a study, allowing for tailored assessments of dementia knowledge. For instance, when including a dementia knowledge survey within a larger questionnaire, a more compact design may be necessary. Techniques such as reusing the subdomain structure of existing scales can be effective in these cases [31]. In this scenario, the internal structure of the instrument remains intact but the number of the questions within each section or the phrasing of the questions may be altered. A similar is approach preselecting questions from an existing instrument to create a more compact representation [32]. Both of these approaches utilize an existing instrument by significantly augmenting its structure or content which may distort the internal properties of the scale and lead to drift from its measured construct.

Another approach is adapting existing instruments according to the needs of a study [33, 34]. In such cases, small augmentations are introduced, but the overall structure and content of the instruments remain intact. While modifications might seem minor, they can impact the psychometric properties of the scale. Thus, caution is warranted when making adjustments.

Although modifying existing instruments can be a straightforward solution, it’s essential to recognize that their psychometric properties and overall effectiveness may differ significantly. This raises questions about data compatibility, as the constructs measured might not align between the adapted and original scales.

#### Designing custom instruments for surveying dementia knowledge

In studies targeting specific populations, researchers often create custom instruments tailored to their sample’s characteristics. Custom instruments typically employ a criterion-referenced strategy, using items drawn from existing literature and previously validated scales. While the resulting items may lack high internal reliability, this strategy allows for flexibility in assessing knowledge on specific topics. For example, Jang et al. created a dementia knowledge instrument for Korean American elders by combining questions from several established instruments [35]. The same approach was utilized by Isaac et al. [36] in researching dementia knowledge among adolescent students, and by Arai et al. [37] and Zülke et al. [38].

Another approach to creating custom scales is generating custom items on face validity [39–44]. However, generating items based on face validity can have drawbacks. The process often lacks systematic evaluation, which can be mitigated by involving a broader team of stakeholders in content validation [42]. Additionally, if the custom instrument diverges significantly from established measures, the resultant data may be incompatible with previously collected datasets, making comparisons challenging.

#### Using hybrid approaches

While these three strategies can be used independently, hybrid approaches may also offer benefits. For instance, Nielsen and Waldemar [45] supplemented the Dementia Knowledge Questionnaire (DKQ) with two questions from the Alzheimer’s disease Awareness Test (ADAT) to address the stigma associated with Alzheimer’s disease. In Croatia, researchers enhanced the Alzheimer’s Disease Knowledge Scale (ADKS) with extra questions on differential diagnosis, pathogenesis, and epidemiology [46]. Similarly, another work supplemented the Dementia Knowledge Assessment Scale (DKAS) with seventeen questions pertaining to different types of dementia, mild cognitive impairment, genetic risk for Alzheimer’s disease, and the impact of dementia on driving [47].

Lastly, it is important to highlight the role of qualitative approaches in studying dementia knowledge. For example, one study used interviews to gather family members’ perspectives and examine whether they view dementia as a terminal condition [48]. Although qualitative methods are less commonly used, we believe that combining quantitative data with qualitative insights can deepen our understanding of dementia knowledge since this approach offers a direct look at how people perceive and conceptualize the disease.

### Single-domain surveys of dementia knowledge

Single-domain surveys focus on specific areas of dementia knowledge, offering benefits for assessing higher or more specialized knowledge levels. Thus, this approach is particularly popular when targeting non-general samples or surveying non-conventional knowledge.

This section discusses studies that have concentrated on individual knowledge domains or have assigned separate tasks for assessing specific areas.

#### Recognition of dementia symptoms

Recognizing dementia symptoms is crucial for early diagnosis and treatment, leading to better outcomes for patients.

In 2003, P. Werner studied public knowledge of Alzheimer’s disease symptoms and its correlation with help-seeking behavior. Participants rated 15 symptoms on a 5-point Likert scale, distinguishing between Alzheimer’s and depressive symptoms [49]. Another example can be found in a work by Low and Anstey [50] where knowledge about dementia symptoms was assessed by using clinical vignettes. Each vignette variation described a character that had symptoms meeting the Diagnostic and Statistical Manual of Mental Disorders, 4th edition (DSM-IV), criteria for Alzheimer’s disease. The lead character in the vignette was described as having mild (50%) or moderate (50%) symptoms of dementia, and as being either male (50%) or female (50%). Participants’ recognition of dementia was determined by asking them what, if anything, was wrong with the lead character in the vignette. The same vignettes were reused in a following study by Low et al. [51] as well as a replicating study published by Nagel et al. in 2021 [52]. Vignettes were also used by Blay and Peluso [53] in their study on the Brazilian public’s ability to recognize Alzheimer’s disease. A similar approach was used in another study, where participants were asked the following question: “What word or words would you use to describe an older adult experiencing memory loss and difficulties with thinking, problem-solving, and language, so much so it affects their ability to perform everyday activities?” [54].

While clinical vignettes offer a more realistic approach to studying dementia recognition capabilities, note that recognition in real life might be poorer than the reported results from vignette tasks. This might be, at least partially, due to the subtle nature of dementia progression. Indeed, family members may not notice subtle changes in cognitive functioning, or they may often attribute them to normal ageing [25, 55].

#### Recognition of risk factors and dementia causes

Knowledge about dementia risk factors and dementia causes is usually studied by using identification lists. In these tasks, participants are presented with a list of items and asked to rate the contribution of each factor to the development of dementia [50, 52, 53]. There may be variations in the item lists depending on the additional factors of interest. For example, Nagel et al. [52] compiled a list containing true dementia contributors, emerging risk factors, supported by partial evidence and factors without evidence, based on popular beliefs. In their work, Blay and Peluso [53] included several factors related to spiritual beliefs and religion, such as “Lack of faith in God”, “Evil eye”, “Fate”, and Low and Anstey [50] added “Weakness of character” and “Laziness” to measure negative perceptions of persons with dementia. Similarly, others explored beliefs about protective behaviors as a natural continuation to the knowledge about risk factors [39, 56, 57]. Zheng et al. [57] presented a randomized list containing risk factors and protective behaviors and asked the participants to mark which items increase the risk of dementia and which lower the risk. A similar approach was used also by Kjelvik et al. in a study from 2022 [39]. Item lists containing etiological factors, protective and risk factors were used in several other works [54, 58–60].

The great benefit of list-based tasks is that they can accurately reflect the current knowledge about dementia. For instance, while the non-modifiable risk factors for dementia have been firmly established over the years, strong empirical evidence for modifiable risk factors has only been demonstrated in the past ten years, and the list of potential risk factors is ever-growing. The Lancet report, first published in 2017 [61], identified 9 modifiable risk factors for dementia (less education, hypertension, hearing impairment, smoking, obesity, depression, physical inactivity, diabetes, and infrequent social contact), the next revision of the report, published in 2020 [11], added another three factors (excessive alcohol consumption, head injury, and air pollution) and with the recently published report from 2024 [62], the list grew with two new additions (untreated vision loss and high LDL cholesterol), accounting for an up-to-date total of 14 modifiable risk factors.

Reflecting the local cultural beliefs surrounding disease genesis is equally important in identifying misconceptions and knowledge gaps about dementia. A prominent example is a study from 2005, exploring knowledge and attitudes towards mental disorders in Nigeria, according to which 32.3% of the respondents attributed “possession by evil spirits” as a cause of mental disorders. Similar findings were reported by Blay and Peluso [53]. Their study on the Brazilian population reported that 68.8% of the participants agreed that a “lack of faith in God” may be related to Alzheimer’s disease and 25.8% agreed that “evil eye” may be a contributor to Alzheimer’s dementia. Religious explanations about dementia were also reported by some South Asian carers who viewed dementia as demons or God’s punishment [55].

### Factors influencing dementia knowledge

All studies typically collect sociodemographic data, such as age, gender, education, marital status, and income. Research on dementia knowledge follows this standard but often examines additional factors that may influence knowledge levels. Identifying influencers and correlates of dementia knowledge is crucial, as this analysis can reveal groups at risk for low literacy. The following sections discuss additional factors impacting dementia knowledge.

#### Prior experience with dementia

The literature identifies three types of direct experiences with dementia: having a diagnosed with dementia relative, providing care to someone with dementia, and working in a profession involving dementia patients. Some studies use general questions like ‘Have you ever known someone with dementia?’ However, this approach fails to differentiate between relatives and professionals, which is critical as professional experience often correlates with greater dementia knowledge and understanding [13, 47, 57, 60, 63, 64].

#### Sources of information

Dementia knowledge correlates extend beyond sociodemographic factors and personal experience. The sources of information individuals use can significantly affect their dementia knowledge [59, 65], making both the quantity and quality of these sources important to consider.

#### Perceived threat

Perceived threat is another important factor influencing dementia knowledge. Another factor that may be considered is the levels of perceived threat. Stronger concerns about developing dementia may influence levels of knowledge about symptoms, risk factors and protective behaviors. Thus, individuals with stronger concerns about developing dementia may exhibit greater knowledge of symptoms, risk factors, and protective behaviors. However, this awareness does not always translate into proactive preventive actions [34, 66]. Interestingly, this knowledge does not necessarily equate to exercising preventive behavioral practices [66, 67]. Along the same lines, increased emotional stress about the consequences of developing Alzheimer’s disease was found to be associated with increased reports of non-warning symptoms as being symptoms of AD among lay people [49].

#### Other influences on dementia knowledge

Finally, studying subgroups in the population would require considering some additional factors. For example, in studying dementia knowledge among ethnic minorities, a factor that can strongly influence dementia knowledge is acculturation [44, 45, 51]. Similarly, when studying knowledge among the medical workforce, one should consider factors such as length of experience, history of dementia-specific trainings, etc [68, 69].

Overall, sociodemographic and experience-based factors play an important role in identifying knowledge gaps in different populations. However, the level of influence a certain socioeconomic characteristics have on dementia knowledge is expected to vary between samples, especially in the context of intracultural or international comparisons. Thus, the selection of possible knowledge influencing factors should be reflecting the specifics of the studied population.

### Complementary assessments

While knowledge shapes the factual understanding that one has about a disease, attitudes and perceptions are intricately related with the emotional response invoked in individuals. As a result, health-related actions can be framed as a behavioral product of the interplay between emotional responses and factual knowing. This view is very much represented by the Health Belief Model where threat perception plays a critical role in determining the likelihood of adhering to preventative measures. The model postulates that anytime there is an increase in an individual’s self-assessed level of susceptibility, there is an increase in the likelihood that the individual will adopt recommended prevention behaviors [70]. In the light of this view, dementia prevention and help-seeking behaviors should also be considered as a product of the interplay between attitudes and knowledge. Thus, to account for the importance of emotional dispositions, we expand our analysis to include examples of commonly studied aspects of attitudes, dispositions and perceptions of dementia.

Nearly twenty works included some form of attitudes evaluation that was either realized through scales such as the Dementia Attitudes Scale (DAS) [71] and the Approaches To Dementia Questionnaire (ADQ) [72] or through a short set of custom questions [43, 51]. Several studies shortly assessed individual components such as fatalism [73], feelings of shame [35], help-seeking intentions [44, 49], personal autonomy [40] and perceptions about dementia and dementia bearers [40, 53]. Another example comes from a study that used seven questions from the Family Stigma in A Scale (FS-ADS) [74] to assess participants’ images of Alzheimer’s disease (AD) patients (e.g., “An AD patient looks untidy”), emotional attributions (e.g., “I would feel shameful if my family had an AD relative”), and social interaction (e.g., “I would avoid social contact with an AD patient”) [73].

Instead of a question-based approach, some studies opted for more descriptive assessment methods. For example, one study assessed negative and positive attributions of dementia by presenting a list of thirteen descriptive terms (unpredictability, fun, gentility, fear, confusion, lost, kind, dangerous, trapped, happy, angry, sad, pathetic or other—each allowing only yes/no (0/1) responses) and asked participants to mark which of these words appear most representative for people who has had dementia for a long time [40].

The prominent use of evaluations of attitudes towards dementia confirms the importance of considering emotional imprints as a driver of health-related behaviors and caretaking practices. Furthermore, the demonstrated variety of studied aspects suggests that emotional dispositions are a complex aspect of health-related behaviors that cannot be treated as a single phenomenon. Thus, more studies should be conducted to uncover the intricate connections between emotions, perceptions, knowledge and action towards dementia.

### A framework for studying dementia knowledge

**Fig. 5:**
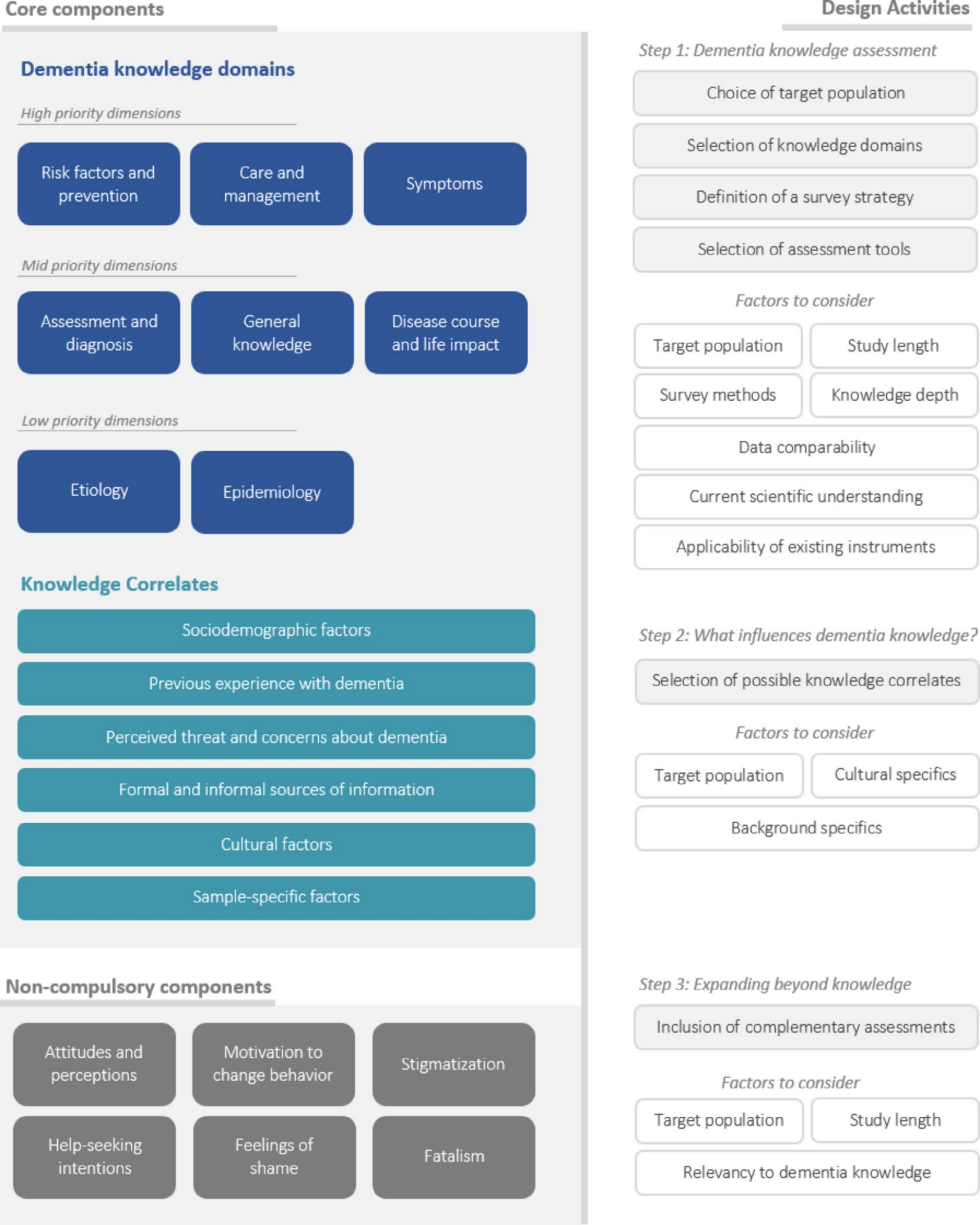
Schematic representation of the proposed framework for studying dementia knowledge. The framework consists of two main components – domains of dementia knowledge and the possible correlates of knowledge that should be considered in a study. We identified eight knowledge domains and assigned them priority levels based on their societal relevance. Additionally, we identified several complimentary assessments that may be considered for inclusion in dementia knowledge studies. While the complimentary components do not directly reflect the levels of literacy, they give information about the personal perceptions and attitudes the society projects toward the disease and those affected by it. The framework also includes a set of activities, developed to guide the process of study planning. The activities describe how the components should be operationalized

Based on the insights presented thus far, a cohesive framework for studying dementia knowledge was established. The proposed framework contains three major components: two core components (dementia knowledge domains, knowledge correlates), compulsory in nature and a complimentary component. Additionally, the framework offers a synthesized list of activities guiding the process of component operationalization (Fig. 5).

#### Eight domains of knowledge

The literature identified eight key domains of dementia knowledge, with the most researched being risk factors and prevention, care and management, and disease symptoms. These findings indicate that studies aim to evaluate public understanding of dementia management and prevention. Understanding risk factors enables individuals to adopt healthier lifestyles while recognizing symptoms is crucial for early treatment access. Finally, understanding the needs and behaviors of sick individuals is a critical part of the caretaking process. Notably, caregivers often face emotional burdens when lacking knowledge about patients’ needs and behaviors, leading to frustration and stress [75]. As a result, these three knowledge domains are highly valuable for research in the general population, as they reflect everyday behaviors and are crucial for guiding educational efforts. Despite etiology and epidemiology being less significant in this context, they gain relevance in studies involving medical specialists.

#### Prioritizing knowledge domains

The identified trends indicate that knowledge domains should be prioritized based on their potential to enhance prevention, early diagnosis, and effective care among the general population. The proposed framework reflects this trend by assigning priority levels to the knowledge domains (Fig. 4). As a result, ‘risk factors and prevention,’ ‘care and management,’ and ‘symptoms’ are designated as high-priority domains, while ‘etiology’ and ‘epidemiology’ are considered low priority. The remaining domains are classified as mid-priority. Importantly, these priority assignments apply to studies of the general population; they may not hold for more knowledgeable samples, such as healthcare professionals or medical students.

#### Considering outside factors influencing knowledge

The current framework features dementia knowledge correlates as an integral part of each dementia knowledge study. Consequently, the framework outlines the most commonly implicated factors, influencing dementia knowledge according to the literature - sociodemographic factors, personal experience, affective factors, cultural factors, and informational sources. By including “sample-specific factors” we are acknowledging the non-exhaustive nature of the provided list while also encouraging authors to consider what other factors may influence the observed knowledge levels of their samples.

#### Complementing dementia knowledge studies

The complementary module is representative of attitudes and perceptions that may be consequential or related to the levels of knowledge. For instance, stigma is defined as an undesired difference that is related to insufficient or inadequate knowledge and often leads to prejudice and discrimination [76]. In the case of dementia, negative attitudes and dementia-related stigma act as a source of social isolation, social exclusion, and, therefore, distress. Furthermore, social stigma creates barriers to help-seeking and effectively delays dementia diagnosis and treatment [77]. Dementia-related stigma can manifest itself in many ways. Prominent examples are - a lack of priority given to the individual’s quality of life because of their dementia diagnosis or a belief that the lack of a cure for dementia means that it should not be an area of attention for highly-skilled medical interventions and research funding [78]. Therefore, the study of attitudes and perceptions of dementia is considered a complementing aspect of knowledge that should not be underestimated, as negative perceptions tend to evolve in discriminatory patterns that can ultimately affect millions of lives.

#### Planning a study

The formulated framework offers a compact action list designed to navigate the planning stages of dementia knowledge studies. A useful way of thinking about the proposed framework is considering the components of the framework as building blocks and the provided action list as a manual for piecing the blocks and creating a study design. The provided list ensures that the selected research methodology is compatible with the specific needs and characteristics of the study’s population and goals.

Although the list may not be exhaustive, it highlights critical moments in study design, safeguarding against planning-stage errors and unintentionally introduced data bias. The proposed action list offers a remedy to the pitfalls of preexisting methodological variabilities by delivering a structured approach to planning that can improve the quality of research in the domain of dementia literacy.

## Discussion

This study offers an overview of global research on dementia knowledge, synthesizing the insights into a unified framework for dementia knowledge exploration. The analysis discusses the strengths and weaknesses of various approaches to studying dementia knowledge, revealing significant variability in assessment methods. The findings underscored the necessity for a unified framework that addresses key aspects of dementia knowledge and provides comprehensive guidance for surveying awareness in this field.

The proposed framework includes two core components—knowledge domains and knowledge correlates—along with a complementary component and an action list to guide the planning of dementia studies. Its goal is to establish a foundational base for future research, support inter-study comparisons, and deepen our understanding of dementia knowledge and attitudes across communities.

### Existence of methodological variability

The literature analysis confirmed the presence of methodological differences across studies, including variability in knowledge domains, assessment methods, and the influences on knowledge and perceptions. These differences often stem from the distinct goals of the studies. For example, studies focused on specialized groups may use general assessment tools, which can lead to ceiling effects. In such cases, customized assessment methods may be more effective.

While we acknowledge that some methodological variability is inevitable, we recommend a structured planning approach to ensure a focused and coherent dementia knowledge research agenda. The proposed framework serves as a roadmap to help researchers select appropriate methodologies aligned with their study objectives.

### Framework components

Eight key domains of dementia knowledge were identified: general knowledge, etiology, epidemiology, disease course and life impact, assessment and diagnosis, symptoms, care and management, and risk factors and protective behaviors. Among these, risk factors and protective behaviors, care and management, and symptoms were the most frequently studied domains. This suggests a focus on aspects of dementia that have immediate implications for prevention, early detection, and patient care. Conversely, etiology and epidemiology were the least studied, reflecting perhaps lesser-perceived importance for public education but a critical area for medical professionals. While these observations cannot be generalized, the validity of the identified knowledge domains is supported by partial overlaps with multiple knowledge scales [13, 26, 28].

The proposed framework draws attention to the critical importance of considering knowledge correlates in conducting well-rounded and informative studies. Therefore, several important factors that may contribute to the levels of dementia knowledge were distinguished: sociodemographic factors, previous experience with dementia, properties of used informational sources, and concerns about developing the disease. Identifying less knowledgeable groups or groups susceptible to misconceptions and false beliefs is critical in designing educational campaigns, thus careful consideration of possible characteristics that may influence the levels of knowledge is central in designing informative studies of dementia knowledge. Considering that the highest benefit of such studies is the impact they may have on educational outreach and informational campaigns, study designs should be carefully crafted to capture the current state of knowledge and understanding as accurately as possible.

Finally, assessments of attitudes, perceptions, and emotionally driven responses to dementia are included as part of the complementary component. While it is considered non-compulsory, the importance of studying non-factual aspects of beliefs and understandings remains highly important as the connection between knowledge, attitudes, perceptions, and actions is unquestionable but still poorly understood. Thus, we include these assessments as a vital component that would increase the value of every dementia knowledge study.

### Framework activities

The proposed framework features two types of structures – components and activities. The readers are invited to think of the components as building blocks and of the activities as an assembly manual. Thus, the proposed framework is applied by executing the activities in order to make an appropriate selection of components according to the needs of a particular study.

Although the list of activities may not be exhaustive, it highlights critical elements in study design, safeguarding against planning-stage errors and unintentionally introduced data bias. For instance, selecting an assessment method for the chosen knowledge domains is a complex task that involves considering multiple factors such as target population, study length, required depth of knowledge, data acquisition methods, the relevance of validated scales, and data interoperability. Studies by Carpenter et al. [13], Spector et al. [79], and others have highlighted that outdated tools can lead to gaps in knowledge assessment and misinformed educational efforts, thus impacting dementia care practices negatively [5, 13, 27, 79]. Similarly, our literature exploration showed variability between domain coverage of existing instruments that is consistent with previous research [12], underscoring the importance of a structured approach to study design and selection of knowledge assessment tools to ensure accurate, comprehensive, and relevant data collection.

### A holistic approach to dementia knowledge

By extending the framework to include components dedicated to possible correlates of dementia knowledge and complementary assessments of attitudes, we aim to underline that knowledge does not exist on its own; it is often driven by personal interests and beliefs as well as environmental, social, and economic factors. Thus, we take a holistic perspective on dementia knowledge and call for a transition towards in-depth studies on health literacy that go beyond the evaluation of factual fluency.

While the formalization of the presented framework is based on an observational account of existing literature, the idea of approaching knowledge in a more holistic way is represented in some theoretical accounts. In particular, the Common-Sense Model (CSM) [80], a theoretical model of how individuals develop and use mental schemata in managing health and illness, identifies three main sources of knowledge influence: personal experience with the illness, external information from formal sources, and informal information from cultural beliefs or social interactions. This overlap highlights the congruence between the CSM and the conceptual foundation of the proposed framework. Furthermore, the CMS suggests that people naturally regulate their behavior to avoid the adverse effects of a health condition, relying on their beliefs and knowledge in the following domains: specific symptoms and the ability to recognize them as an illness, consequences or impact of the illness on one’s life, timeline of the illness, causes, perceptions of the controllability or curability of the illness and emotional responses to the illness. These dimensions closely match the identified domains of dementia knowledge. Thus, underlining the validity of the framework and the significance of the present work.

### Implications for Research and Practice

The proposed framework offers a structured approach to studying dementia knowledge and attitudes, which can improve the comparability and generalizability of future research findings based on surveys. By prioritizing knowledge domains that are directly translatable to prevention, early diagnosis, and effective management, researchers and authorities can focus on areas that have the greatest potential to reduce the societal burden of dementia. Furthermore, understanding the correlates of dementia knowledge can inform the development of targeted educational programs, tailored to address specific knowledge gaps in different demographic groups.

The inclusion of complementary assessments of attitudes and stigma is particularly important. Addressing these issues through public education campaigns can reduce the social isolation and emotional distress experienced by dementia patients and their caregivers [21]. Reducing stigma can also encourage early help-seeking behaviors and improve the overall quality of life for individuals with dementia [81].

## Limitations and future work

The present work should be interpreted in the light of several limitations.

First, since the proposed framework is a product of a narrative review, it bears some of the limitations stemming from it. For instance, one could argue that the framework is based on a non-systematic approach of literature review thus skewing the current state of research on dementia knowledge. Indeed while narrative reviews use comprehensive strategies for literature search they are less exhaustive than systematic reviews and more prone to bias. Nevertheless, we took a structured approach to literature search, complying with the PRISMA guidelines, in order to provide an additional level of transparency to our methodology. Furthermore, we believe that the literature sample used in the present work is sufficient to represent the tendencies and dynamics in dementia knowledge research as it offers a high level of internal variability. In particular, the collected studies represent a sufficiently large period of time and have a wide geographical coverage. Similarly, our sample includes studies featuring general populations, ethnic and specialized samples, and older and younger adults, thus capturing methodological intricacies that may arise from specific sample characteristics. Therefore, the resulting framework is applicable to various dementia knowledge studies utilizing structured questionnaires. Of course, this remains to be formally justified by future studies applying the framework to their work.

Next, despite acquiring a diverse literature sample our literature search was limited to English and did not take into account research studies in other languages. As a result, it may be the case that relevant literature was not considered in the present study. Future research should consider expanding the topic beyond English, as dementia is a global issue that should be explored on an international level.

Next, while the present work offers a theoretical description of the framework and justification for its conception, it does not provide a practical account of its application. While we acknowledge this limitation, it is important to note that we chose to develop a separate case study that demonstrates how the framework is applied to a real dementia knowledge study. Currently a work in progress, this extension will explore the practical dimensions of applying the framework as a planning tool. Thus, future work will focus on applying the framework in practice and conducting a study on dementia knowledge among the general population of Bulgaria.

## Conclusions

This study proposes a common, unified framework for future studies surveying dementia awareness at a broad community level. By taking a global perspective on dementia knowledge and putting it in the context of its determinants, the framework aims to support the comparability of research findings and inform the development of targeted educational initiatives. Thus, the proposed framework plays a crucial role in addressing knowledge gaps, misconceptions, and stigma about dementia - essential steps in mitigating the societal impact of dementia and improving the lives of those affected by the disease.

## Electronic Supplementary Material

Below is the link to the electronic supplementary material.

Additional file 1: Full list of literature used to compile the framework

## Data Availability

No datasets were generated or analysed during the current study.

## Abbreviations

ADKS: Alzheimer’s Disease Knowledge Scale
DKAS: Dementia Knowledge Assessment Scale
DKQ: Dementia Knowledge Questionnaire
DKAT2: Dementia Knowledge Assessment Tool
DSM: IV-Diagnostic and Statistical Manual of Mental Disorders, 4th edition
ADAT: Alzheimer’s Disease Awareness Test
DAS: Dementia Attitudes Scale
ADQ: Approaches To Dementia Questionnaire
FS: ADS-Family Stigma in Alzheimer’s Disease Scale
CSM: Common-Sense Model
