# Supplementary material for: Establishing a unified global framework for studying dementia knowledge: insights from a narrative review
Source: Arch Public Health. 2024 Dec 23;82:242. doi: 10.1186/s13690-024-01476-1 (PMC11665209; doi:10.1186/s13690-024-01476-1)
Supplement: Supplementary file 1 — Additional file 1: Full list of literature used to compile the framework [file 13690_2024_1476_MOESM1_ESM.docx]

*Table S1: List of publications included in the analysis. The list is sorted by year of publishing in a descending order. The "Survey strategy" column describes the methodological approach of each study in examining dementia knowledge (Reusing = reusing existing instruments; Partially reusing = partially reusing existing instruments; Custom = developed a custom instrument for study). The "single-domain tasks" column shows whether the study includes tasks dedicated to examining a single domain of knowledge. Knowledge domains are numerically represented under the following mapping: (1) general knowledge; (2) etiology; (3) epidemiology; (4) disease course and life impact; (5) assessment and diagnosis; (6) symptoms; (7) care and management; (8) risk factors and protective behaviors.*

| Title | Year | Country | Survey Strategy | Single- domain tasks | Domains of knowledge |
| --- | --- | --- | --- | --- | --- |
| Dementia knowledge and attitudes of nursing undergraduate students—association between lifestyle background and practical training experience [1] | 2024 | Japan | Reusing | - | 2,4,6,7 |
| Evaluation of knowledge and attitudes regarding Alzheimer’s disease and related dementia among medical students in Palestine: A cross-sectional study [2] | 2024 | Palestine | Reusing | - | 4,5,6,7,8 |
| Awareness, attitudes, and beliefs of dementia in Indonesia [3] | 2024 | Indonesia | Custom | Symptoms recognition;  Causes of dementia | 2,4,5,6 |
| Dementia awareness in Egypt: what do people really know? [4] | 2024 | Egypt | Custom | - | 1,4,5,6,8 |
| Knowledge and motivation to adopt recommended health behaviours to reduce dementia risk among the elderly in Norway: a qualitative study [5] | 2023 | Norway | Custom | - | 8 |
| Nursing students’ knowledge and attitudes about dementia: A cross-sectional study [6] | 2023 | Turkey | Reusing | - | 1,2,3,4,7,8 |
| Association of Knowledge About Dementia with Two Dimensional Attitudes Among a Community Population in South Korea [7] | 2023 | South Korea | Custom | - | 1,7 |
| Public Knowledge about Dementia in Poland—A Survey Study [8] | 2023 | Poland | Reusing | - | 1,2,3,4,7,8 |
| Knowledge and attitudes of community pharmacists regarding dementia: A nationwide cross‐sectional study in Vietnam [9] | 2023 | Vietnam | Custom | - | 1,6 |
| Public knowledge about dementia risk reduction in Norway [10] | 2022 | Norway | Custom | Risk/protective factors identification | 8 |
| Dementia is (not) a natural part of ageing: a cross-sectional study on dementia knowledge and misconceptions in Swiss and Italian young adults, adults, and older adults [11] | 2022 | Switzerland, Italy | Reusing | - | 1,2,3,4,7,8 |
| Perception and knowledge of dementia prevention and its associated socio-demographic factors in China: A community-based cross-sectional study [12] | 2022 | China | Custom | Risk factors identification | 8 |
| Lay Knowledge About Dementia in Iceland: Symptoms and Risk and Protective Factors [13] | 2022 | Iceland | Partially reusing | Symptoms recognition;  Risk/protective factors identification | 6,8 |
| Knowledge, Attitudes, and Perceptions Toward Dementia Among Middle-Aged Singapore Residents [14] | 2022 | Singapore | Custom | - | 1,6,7,8 |
| The attitude and knowledge of medical students regarding dementia [15] | 2022 | Croatia | Hybrid | - | 2,3,4,5,6,7,8 |
| Knowledge of risk and protective factors for dementia in older German adults: A  population-based survey on risk and protective factors for dementia and internet based  brain health interventions [16] | 2022 | Germany | Custom | Risk/protective factors | 8 |
| Dementia-Preventing Behavior Awareness and Uptake Rates among Japanese Women in Midlife: A Survey-Based Pilot Study [17] | 2022 | Japan | Reusing | - | 2,4,6,7 |
| How do factors of sociodemographic, health literacy and dementia experience influence carers’ knowledge of dementia? [18] | 2022 | UK | Reusing | - | 1,2,3,4,7,8 |
| Knowledge and perceptions of Alzheimer’s disease in three ethnic groups of younger adults in the United Kingdom [19] | 2021 | UK | Reusing | - | 4,5,6,7,8 |
| Knowledge and attitudes in dementia held by general practitioners in the primary care setting of Botucatu, São Paulo, Brazil [20] | 2021 | Brazil | Custom | - | 3,4,7 |
| Factors Associated With Dementia Knowledge and Dementia Worry in the South Korean Elderly Population [21] | 2021 | South Korea | Partially reusing | - | 2,4,6,7 |
| What do the public really know about dementia and its risk factors? [22] | 2021 | Australia | Custom | Symptoms recognition;  Risk factors identification | 6,8 |
| Knowledge, health beliefs and attitudes towards dementia and dementia risk reduction among the Dutch general population: a cross-sectional study [23] | 2021 | Netherlands | Reusing | - | 1,2,3,4,7,8 |
| A Survey of Dementia Knowledge and Recognition of Dementia Prevention and Practice in Healthy Older Adults [24] | 2021 | Japan | Reusing | - | 2,4,6,7 |
| Public awareness, knowledge, and attitude toward Alzheimer’s disease in Aseer region, Saudi Arabia [25] | 2020 | Saudi Arabia | Reusing | - | 4,5,6,7,8 |
| Assessing Knowledge and Perceptions of Alzheimer’s Disease Among Employees of a Pharmaceutical Company in Spain: A Comparison Between Caregivers and Non-Caregivers [26] | 2020 | Spain | Reusing | - | 4,5,6,7,8 |
| Knowledge of Dementia Among the Australian Health Workforce: A National Online Survey [27] | 2020 | Australia | Reusing | - | 1,2,3,4,7,8 |
| Knowledge regarding Alzheimer’s Disease among College Students of Kathmandu, Nepal [28] | 2020 | Nepal | Reusing | - | 4,5,6,7,8 |
| Alzheimer’s disease knowledge among American Indians and Alaska Natives [29] | 2020 | USA | Reusing | - | 4,5,6,7,8 |
| Public knowledge of late-life cognitive decline and dementia in an international sample [30] | 2020 | International | Hybrid | - | 1,2,3,4,7,8 |
| Public awareness and knowledge of factors associated with dementia in China [31] | 2020 | China | Custom | Risk/protective factors identification | 8 |
| Level of Knowledge About Alzheimer’s Disease Among Nursing Staff in Suzhou and its Influencing Factors [32] | 2019 | China | Reusing | - | 4,5,6,7,8 |
| Dementia awareness and risk perception in middle-aged and older individuals: baseline results of the MijnBreincoach survey on the association between lifestyle and brain health [33] | 2019 | Netherlands | Custom | Risk/protective factors identification | 8 |
| Factors associated with public knowledge of and attitudes to dementia: A cross-sectional study [34] | 2019 | Ireland | Custom | - | 1,8 |
| Knowledge of Dementia: Do family members understand dementia as a terminal condition? [35] | 2017 | Australia | Qualitative approach | - | - |
| Public knowledge and understanding of dementia —evidence from a national survey in Ireland [36] | 2017 | Ireland | Custom | - | 1,3,8 |
| Dementia Literacy among Community-Dwelling Older Adults in Urban China: A Cross-sectional Study [37] | 2017 | China | Partially reusing | - | 1,3,4,7 |
| Knowledge and attitudes towards dementia in adolescent students [38] | 2017 | UK | Custom | - | 1,2,3,4,7 |
| Public knowledge about dementia in Germany —results of a population survey [39] | 2016 | Germany | Partially reusing | - | 1,5,6,7,8 |
| Knowledge and perceptions of dementia and Alzheimer’s disease in four ethnic groups in Copenhagen, Denmark: Knowledge of dementia in four ethnic groups [40] | 2016 | Denmark | Hybrid | - | 1,2,3,6,8 |
| Public knowledge about dementia in South Korea: a community-based cross-sectional survey [41] | 2015 | South Korea | Partially reusing | - | 2,4,6,7 |
| Perception of Alzheimer’s disease in the French population [42] | 2014 | France | Custom | - | 1,6,7,8 |
| Gaps in Alzheimer’s Knowledge Among College Students [43] | 2014 | USA | Reusing | - | 4,5,6,7,8 |
| Public beliefs and knowledge about risk and protective factors for Alzheimer’s disease [44] | 2014 | USA | Custom | Risk/protective factors identification | 8 |
| Who Knows, who Cares? Dementia Knowledge among Nurses, Care Workers, and Family members of People Living with Dementia [45] | 2014 | Australia | Reusing | - | 1;2;3;4;6;7 |
| Levels and Correlates of Knowledge about Alzheimer’s Disease among Older Chinese Americans [46] | 2014 | USA | Partially reusing | - | 2,3,4,5,6,7,8 |
| Nursing students’ knowledge and attitudes towards dementia — A questionnaire survey [47] | 2013 | Malta | Reusing | - | 4,5,6,7,8 |
| The Impact of Experience with a Family Member with Alzheimer’s Disease on Views about the Disease across Five Countries [48] | 2012 | Germany, Spain, USA, Poland, France | Custom | - | 5,6,7 |
| Beliefs about Alzheimer’s disease in Britain [49] | 2012 | UK | Reusing | - | 4,5,6,7,8 |
| Dementia knowledge and attitudes of the general public in Northern Ireland: an analysis of national survey data [50] | 2012 | Ireland | Custom | - | 1,7,8 |
| Demographic and Contextual Factors Related to Knowledge About Alzheimer’s Disease [51] | 2011 | USA | Reusing | - | 4,5,6,7,8 |
| Recognition, Attitudes and Causal Beliefs regarding Dementia in Italian, Greek and Chinese Australians [52] | 2010 | Australia | Custom | Symptoms recognition;  Risk factors identification | 1,8 |
| Knowledge of Alzheimer’s Disease, Feelings of Shame, and Awareness of Services Among Korean American Elders [53] | 2010 | USA | Custom | - | 1,3,4,5,6,7 |
| Dementia literacy: Recognition and beliefs on dementia of the Australian public [54] | 2009 | Australia | Custom | Symptoms recognition;  Risk factors identification | 1,8 |
| What do we know about dementia?: a survey on knowledge about dementia in the general public of Japan [55] | 2008 | Japan | Custom | - | 1,4,5,6 |
| The Public’s Ability to Recognize Alzheimer Disease and Their Beliefs About Its Causes [56] | 2008 | Brazil | Custom | Symptoms recognition;  Risk factors identification | 1,6,8 |
| Knowledge of dementia among South Asian (Indian) older people in Manchester, UK [57] | 2007 | UK | Reusing | - | 1,2,3,6,8 |
| Knowledge of Alzheimer’s disease in four ethnic groups of older adults [58] | 2004 | USA | Custom | - | 1,2,4,5,6,7,8 |
| Knowledge about symptoms of Alzheimer’s disease: correlates and relationship to help‐seeking behavior [59] | 2003 | USA | Custom | Symptoms recognition | 6 |

References:

[1] H. Nanbu, K. Hayashi, F. Tanji, Y. Tsuruta, K. Awaji, and N. Nakai, “Dementia knowledge and attitudes of nursing undergraduate students—association between lifestyle background and practical training experience—,” *J Rural Med*, vol. 19, no. 2, pp. 83–91, 2024, doi: 10.2185/jrm.2023-042.

[2] M. Abuawad *et al.*, “Evaluation of knowledge and attitudes regarding Alzheimer’s disease and related dementia among medical students in Palestine: A cross-sectional study,” *PLoS ONE*, vol. 19, no. 5, p. e0304012, May 2024, doi: 10.1371/journal.pone.0304012.

[3] N. Farina *et al.*, “Awareness, attitudes, and beliefs of dementia in Indonesia,” *Alz & Dem Diag Ass & Dis Mo*, vol. 16, no. 2, p. e12570, Apr. 2024, doi: 10.1002/dad2.12570.

[4] A. A. Ashour *et al.*, “Dementia awareness in Egypt: what do people really know?,” *Egypt J Neurol Psychiatry Neurosurg*, vol. 60, no. 1, p. 91, Jul. 2024, doi: 10.1186/s41983-024-00864-2.

[5] G. Kjelvik, G. Selbæk, and A. M. M. Rokstad, “Knowledge and motivation to adopt recommended health behaviours to reduce dementia risk among the elderly in Norway: a qualitative study,” *Public Health*, vol. 221, pp. 60–65, Aug. 2023, doi: 10.1016/j.puhe.2023.06.002.

[6] G. Korkmaz Aslan, E. Kılınç İşleyen, and A. Kartal, “Nursing students’ knowledge and attitudes about dementia: A cross-sectional study,” *Nurse Education in Practice*, vol. 72, p. 103800, Oct. 2023, doi: 10.1016/j.nepr.2023.103800.

[7] S. Lee *et al.*, “Association of Knowledge About Dementia with Two Dimensional Attitudes Among a Community Population in South Korea,” *JAD*, vol. 92, no. 2, pp. 565–572, Mar. 2023, doi: 10.3233/JAD-220736.

[8] A. Skowronek, K. Bojkowska-Otrębska, and B. Łabuz-Roszak, “Public Knowledge about Dementia in Poland—A Survey Study,” *JCM*, vol. 12, no. 24, p. 7675, Dec. 2023, doi: 10.3390/jcm12247675.

[9] H. T. Thanh Nguyen and D. X. Dinh, “Knowledge and attitudes of community pharmacists regarding dementia: A nationwide cross‐sectional study in Vietnam,” *Int J Geriat Psychiatry*, vol. 38, no. 8, p. e5981, Aug. 2023, doi: 10.1002/gps.5981.

[10] G. Kjelvik *et al.*, “Public knowledge about dementia risk reduction in Norway,” *BMC Public Health*, vol. 22, no. 1, p. 2046, Nov. 2022, doi: 10.1186/s12889-022-14433-w.

[11] D. Pacifico *et al.*, “Dementia is (not) a natural part of ageing: a cross-sectional study on dementia knowledge and misconceptions in Swiss and Italian young adults, adults, and older adults,” *BMC Public Health*, vol. 22, no. 1, p. 2176, Nov. 2022, doi: 10.1186/s12889-022-14578-8.

[12] D. Song, D. Yu, and Q. Sun, “Perception and knowledge of dementia prevention and its associated socio-demographic factors in China: A community-based cross-sectional study,” *Front. Neurosci.*, vol. 16, p. 1093169, Dec. 2022, doi: 10.3389/fnins.2022.1093169.

[13] M. K. Jónsdóttir, E. B. Pálsdóttir, S. Ý. Hannesdóttir, and T. Karlsson, “Lay Knowledge About Dementia in Iceland: Symptoms and Risk and Protective Factors,” *Gerontology and Geriatric Medicine*, vol. 8, p. 233372142211429, Jan. 2022, doi: 10.1177/23337214221142937.

[14] F. Siddiqui *et al.*, “Knowledge, Attitudes, and Perceptions Toward Dementia Among Middle-Aged Singapore Residents,” *JAD*, vol. 86, no. 1, pp. 231–244, Mar. 2022, doi: 10.3233/JAD-215262.

[15] J. Stojic, M. Petrosanec, M. Milosevic, and M. Boban, “The attitude and knowledge of medical students regarding dementia,” *Acta Neurol Belg*, vol. 122, no. 3, pp. 625–630, Jun. 2022, doi: 10.1007/s13760-022-01939-8.

[16] A. E. Zülke, M. Luppa, S. Köhler, and S. G. Riedel-Heller, “Knowledge of risk and protective factors for dementia in older German adults A population-based survey on risk and protective factors for dementia and internet-based brain health interventions,” *PLoS ONE*, vol. 17, no. 11, p. e0277037, Nov. 2022, doi: 10.1371/journal.pone.0277037.

[17] Y. Suzuki, N. Yamane, K. Tsukagoshi, M. Yamaguchi, and H. Mochizuki, “Dementia-Preventing Behavior Awareness and Uptake Rates among Japanese Women in Midlife: A Survey-Based Pilot Study,” *IJERPH*, vol. 19, no. 16, p. 10029, Aug. 2022, doi: 10.3390/ijerph191610029.

[18] S. Crawley, K. Moore, V. Vickerstaff, E. Fisher, C. Cooper, and E. L. Sampson, “How do factors of sociodemographic, health literacy and dementia experience influence carers’ knowledge of dementia?,” *Dementia*, vol. 21, no. 4, pp. 1270–1288, May 2022, doi: 10.1177/14713012221074219.

[19] A. H. Kafadar, C. Barrett, and K. L. Cheung, “Knowledge and perceptions of Alzheimer’s disease in three ethnic groups of younger adults in the United Kingdom,” *BMC Public Health*, vol. 21, no. 1, p. 1124, Dec. 2021, doi: 10.1186/s12889-021-11231-8.

[20] V. F. D. S. Mayoral, P. J. F. Villas Boas, and A. F. Jacinto, “Knowledge and attitudes in dementia held by general practitioners in the primary care setting of Botucatu, São Paulo, Brazil,” *Arq. Neuro-Psiquiatr.*, vol. 79, no. 2, pp. 107–113, Feb. 2021, doi: 10.1590/0004-282x-anp-2020-0051.

[21] S. H. Joo, I. S. Jo, H. J. Kim, and C. U. Lee, “Factors Associated With Dementia Knowledge and Dementia Worry in the South Korean Elderly Population,” *Psychiatry Investig*, vol. 18, no. 12, pp. 1198–1204, Dec. 2021, doi: 10.30773/pi.2021.0295.

[22] A. K. Nagel, T. Loetscher, A. E. Smith, and H. A. Keage, “What do the public really know about dementia and its risk factors?,” *Dementia*, vol. 20, no. 7, pp. 2424–2440, Oct. 2021, doi: 10.1177/1471301221997301.

[23] J. Vrijsen, T. F. Matulessij, T. Joxhorst, S. E. de Rooij, and N. Smidt, “Knowledge, health beliefs and attitudes towards dementia and dementia risk reduction among the Dutch general population: a cross-sectional study,” *BMC Public Health*, vol. 21, no. 1, p. 857, Dec. 2021, doi: 10.1186/s12889-021-10913-7.

[24] N. Yamane, K. Tsukagoshi, M. Hisada, M. Yamaguchi, and Y. Suzuki, “A Survey of Dementia Knowledge and Recognition of Dementia Prevention and Practice in Healthy Older Adults,” *Dement Geriatr Cogn Disord Extra*, vol. 11, no. 3, pp. 256–263, Nov. 2021, doi: 10.1159/000519513.

[25] A. A. Alhazzani, A. M. Alqahtani, M. S. Alqahtani, T. M. Alahmari, and A. A. Zarbah, “Public awareness, knowledge, and attitude toward Alzheimer’s disease in Aseer region, Saudi Arabia,” *Egypt J Neurol Psychiatry Neurosurg*, vol. 56, no. 1, p. 81, Dec. 2020, doi: 10.1186/s41983-020-00213-z.

[26] G. Garcia-Ribas, E. García-Arcelay, A. Montoya, and J. Maurino, “Assessing Knowledge and Perceptions of Alzheimer’s Disease Among Employees of a Pharmaceutical Company in Spain: A Comparison Between Caregivers and Non-Caregivers,” *PPA*, vol. Volume 14, pp. 2357–2364, Dec. 2020, doi: 10.2147/PPA.S282147.

[27] M. J. Annear, “Knowledge of Dementia Among the Australian Health Workforce: A National Online Survey,” *J Appl Gerontol*, vol. 39, no. 1, pp. 62–73, Jan. 2020, doi: 10.1177/0733464817752085.

[28] K. Baral, M. Dahal, and S. Pradhan, “Knowledge regarding Alzheimer’s Disease among College Students of Kathmandu, Nepal,” *International Journal of Alzheimer’s Disease*, vol. 2020, pp. 1–6, Jan. 2020, doi: 10.1155/2020/6173217.

[29] M. Jernigan, A. D. Boyd, C. Noonan, and D. Buchwald, “Alzheimer’s disease knowledge among American Indians and Alaska Natives,” *A&D Transl Res & Clin Interv*, vol. 6, no. 1, p. e12101, Jan. 2020, doi: 10.1002/trc2.12101.

[30] R. Van Patten and G. Tremont, “Public knowledge of late-life cognitive decline and dementia in an international sample,” *Dementia*, vol. 19, no. 6, pp. 1758–1776, Aug. 2020, doi: 10.1177/1471301218805923.

[31] Y.-B. Zheng *et al.*, “Public awareness and knowledge of factors associated with dementia in China,” *BMC Public Health*, vol. 20, no. 1, p. 1567, Dec. 2020, doi: 10.1186/s12889-020-09665-7.

[32] L. Lin, S. Lv, J. Liang, H. Li, and Y. Xu, “Level of Knowledge About Alzheimer’s Disease Among Nursing Staff in Suzhou and its Influencing Factors,” *CAR*, vol. 16, no. 7, pp. 650–658, Sep. 2019, doi: 10.2174/1567205016666190726102935.

[33] I. Heger *et al.*, “Dementia awareness and risk perception in middle-aged and older individuals: baseline results of the MijnBreincoach survey on the association between lifestyle and brain health,” *BMC Public Health*, vol. 19, no. 1, p. 678, Dec. 2019, doi: 10.1186/s12889-019-7010-z.

[34] M. Rosato, G. Leavey, J. Cooper, P. De Cock, and P. Devine, “Factors associated with public knowledge of and attitudes to dementia: A cross-sectional study,” *PLoS ONE*, vol. 14, no. 2, p. e0210543, Feb. 2019, doi: 10.1371/journal.pone.0210543.

[35] S. Andrews, F. McInerney, C. Toye, C.-A. Parkinson, and A. Robinson, “Knowledge of Dementia: Do family members understand dementia as a terminal condition?,” *Dementia*, vol. 16, no. 5, pp. 556–575, Jul. 2017, doi: 10.1177/1471301215605630.

[36] R. W. Glynn, E. Shelley, and B. A. Lawlor, “Public knowledge and understanding of dementia—evidence from a national survey in Ireland,” *Age and Ageing*, vol. 46, no. 5, pp. 865–869, Sep. 2017, doi: 10.1093/ageing/afx082.

[37] H. Zhang *et al.*, “Dementia Literacy among Community-Dwelling Older Adults in Urban China: A Cross-sectional Study,” *Front. Public Health*, vol. 5, p. 124, Jun. 2017, doi: 10.3389/fpubh.2017.00124.

[38] M. G. E. K. N. Isaac, M. M. Isaac, N. Farina, and N. Tabet, “Knowledge and attitudes towards dementia in adolescent students,” *Journal of Mental Health*, vol. 26, no. 5, pp. 419–425, Sep. 2017, doi: 10.1080/09638237.2016.1207234.

[39] D. Lüdecke, O. von dem Knesebeck, and C. Kofahl, “Public knowledge about dementia in Germany—results of a population survey,” *Int J Public Health*, vol. 61, no. 1, pp. 9–16, Jan. 2016, doi: 10.1007/s00038-015-0703-x.

[40] T. R. Nielsen and G. Waldemar, “Knowledge and perceptions of dementia and Alzheimer’s disease in four ethnic groups in Copenhagen, Denmark: Knowledge of dementia in four ethnic groups,” *Int J Geriatr Psychiatry*, vol. 31, no. 3, pp. 222–230, Mar. 2016, doi: 10.1002/gps.4314.

[41] H.-J. Seo, D. Y. Lee, and M. R. Sung, “Public knowledge about dementia in South Korea: a community-based cross-sectional survey,” *Int. Psychogeriatr.*, vol. 27, no. 3, pp. 463–469, Mar. 2015, doi: 10.1017/S1041610214001896.

[42] A. Breining *et al.*, “Perception of Alzheimer’s disease in the French population,” *J Nutr Health Aging*, vol. 18, no. 4, pp. 393–399, Apr. 2014, doi: 10.1007/s12603-014-0008-4.

[43] E. M. Eshbaugh, “Gaps in Alzheimer’s Knowledge Among College Students,” *Educational Gerontology*, vol. 40, no. 9, pp. 655–665, Sep. 2014, doi: 10.1080/03601277.2013.863573.

[44] J. S. Roberts, S. J. McLaughlin, and C. M. Connell, “Public beliefs and knowledge about risk and protective factors for Alzheimer’s disease,” *Alzheimer’s &amp; Dementia*, vol. 10, no. 5S, Oct. 2014, doi: 10.1016/j.jalz.2013.07.001.

[45] A. Robinson *et al.*, “Who Knows, who Cares? Dementia Knowledge among Nurses, Care Workers, and Family members of People Living with Dementia,” *J Palliat Care*, vol. 30, no. 3, pp. 158–165, Sep. 2014, doi: 10.1177/082585971403000305.

[46] F. Sun, X. Gao, H. Shen, and D. Burnette, “Levels and Correlates of Knowledge about Alzheimer’s Disease among Older Chinese Americans,” *J Cross Cult Gerontol*, vol. 29, no. 2, pp. 173–183, Jun. 2014, doi: 10.1007/s10823-014-9229-6.

[47] A. Scerri and C. Scerri, “Nursing students’ knowledge and attitudes towards dementia — A questionnaire survey,” *Nurse Education Today*, vol. 33, no. 9, pp. 962–968, Sep. 2013, doi: 10.1016/j.nedt.2012.11.001.

[48] R. J. Blendon *et al.*, “The Impact of Experience with a Family Member with Alzheimer’s Disease on Views about the Disease across Five Countries,” *International Journal of Alzheimer’s Disease*, vol. 2012, pp. 1–9, 2012, doi: 10.1155/2012/903645.

[49] J. M. Hudson, P. M. J. Pollux, B. Mistry, and S. Hobson, “Beliefs about Alzheimer’s disease in Britain,” *Aging & Mental Health*, vol. 16, no. 7, pp. 828–835, Sep. 2012, doi: 10.1080/13607863.2012.660620.

[50] P. McParland, P. Devine, A. Innes, and V. Gayle, “Dementia knowledge and attitudes of the general public in Northern Ireland: an analysis of national survey data,” *Int. Psychogeriatr.*, vol. 24, no. 10, pp. 1600–1613, Oct. 2012, doi: 10.1017/S1041610212000658.

[51] B. D. Carpenter, S. M. Zoller, S. Balsis, P. G. Otilingam, and M. Gatz, “Demographic and Contextual Factors Related to Knowledge About Alzheimer’s Disease,” *Am J Alzheimers Dis Other Demen*, vol. 26, no. 2, pp. 121–126, Mar. 2011, doi: 10.1177/1533317510394157.

[52] L.-F. Low *et al.*, “Recognition, Attitudes and Causal Beliefs regarding Dementia in Italian, Greek and Chinese Australians,” *Dement Geriatr Cogn Disord*, vol. 30, no. 6, pp. 499–508, 2010, doi: 10.1159/000321667.

[53] Yuri Jang, G. Kim, and D. Chiriboga, “Knowledge of Alzheimer’s Disease, Feelings of Shame, and Awareness of Services Among Korean American Elders,” *J Aging Health*, vol. 22, no. 4, pp. 419–433, Jun. 2010, doi: 10.1177/0898264309360672.

[54] L. Low and K. J. Anstey, “Dementia literacy: Recognition and beliefs on dementia of the Australian public,” *Alzheimer’s &amp; Dementia*, vol. 5, no. 1, pp. 43–49, Jan. 2009, doi: 10.1016/j.jalz.2008.03.011.

[55] Y. Arai, A. Arai, and S. H. Zarit, “What do we know about dementia?: a survey on knowledge about dementia in the general public of Japan,” *Int J Geriat Psychiatry*, vol. 23, no. 4, pp. 433–438, Apr. 2008, doi: 10.1002/gps.1977.

[56] S. L. Blay and É. de T. Piza Peluso, “The Public’s Ability to Recognize Alzheimer Disease and Their Beliefs About Its Causes,” *Alzheimer Disease & Associated Disorders*, vol. 22, no. 1, pp. 79–85, Jan. 2008, doi: 10.1097/WAD.0b013e31815ccd47.

[57] N. Purandare, V. Luthra, C. Swarbrick, and A. Burns, “Knowledge of dementia among South Asian (Indian) older people in Manchester, UK,” *Int J Geriat Psychiatry*, vol. 22, no. 8, pp. 777–781, Aug. 2007, doi: 10.1002/gps.1740.

[58] L. Ayalon and P. A. Areán, “Knowledge of Alzheimer’s disease in four ethnic groups of older adults,” *Int. J. Geriat. Psychiatry*, vol. 19, no. 1, pp. 51–57, Jan. 2004, doi: 10.1002/gps.1037.

[59] P. Werner, “Knowledge about symptoms of Alzheimer’s disease: correlates and relationship to help‐seeking behavior,” *Int J Geriat Psychiatry*, vol. 18, no. 11, pp. 1029–1036, Nov. 2003, doi: 10.1002/gps.1011.
